# Adaptor SKAP-55 Binds p21^ras^ Activating Exchange Factor RasGRP1 and Negatively Regulates the p21^ras^-ERK Pathway in T-Cells

**DOI:** 10.1371/journal.pone.0001718

**Published:** 2008-03-05

**Authors:** Helga Schneider, Hongyan Wang, Monika Raab, Elke Valk, Xin Smith, Matt Lovatt, Zhonglin Wu, Braudel Maqueira-Iglesias, Klaus Strebhardt, Christopher E. Rudd

**Affiliations:** 1 Cell Signalling Section, Department of Pathology, University of Cambridge, Cambridge, United Kingdom; 2 Molecular Immunology Section, Division of Investigative Sciences, Faculty of Medicine, Imperial College London, Hammersmith Hospital, London, United Kingdom; 3 Cambridge Institute for Medical Research, Cambridge, United Kingdom; 4 Department of Gynecology and Obstetrics, Medical School, Johann Wolfgang Goethe-University, Frankfurt, Germany; Oregon Health & Science University, United States of America

## Abstract

While the adaptor SKAP-55 mediates LFA-1 adhesion on T-cells, it is not known whether the adaptor regulates other aspects of signaling. SKAP-55 could potentially act as a node to coordinate the modulation of adhesion with downstream signaling. In this regard, the GTPase p21^ras^ and the extracellular signal-regulated kinase (ERK) pathway play central roles in T-cell function. In this study, we report that SKAP-55 has opposing effects on adhesion and the activation of the p21^ras^ -ERK pathway in T-cells. SKAP-55 deficient primary T-cells showed a defect in LFA-1 adhesion concurrent with the hyper-activation of the ERK pathway relative to wild-type cells. RNAi knock down (KD) of SKAP-55 in T-cell lines also showed an increase in p21^ras^ activation, while over-expression of SKAP-55 inhibited activation of ERK and its transcriptional target ELK. Three observations implicated the p21^ras^ activating exchange factor RasGRP1 in the process. Firstly, SKAP-55 bound to RasGRP1 via its C-terminus, while secondly, the loss of binding abrogated SKAP-55 inhibition of ERK and ELK activation. Thirdly, *SKAP-55−/−* primary T-cells showed an increased presence of RasGRP1 in the trans-Golgi network (TGN) following TCR activation, the site where p21^ras^ becomes activated. Our findings indicate that SKAP-55 has a dual role in regulating p21^ras^-ERK pathway via RasGRP1, as a possible mechanism to restrict activation during T-cell adhesion.

## Introduction

Conjugate formation between T cells and antigen-presenting cells (APCs) is mediated by lymphocyte function-associated antigen (LFA)-1 and is accompanied by the rearrangement of receptors at the immunological synapse [1], [2]. This adhesion process is regulated by an array of adaptors that include SLP-76 (76-kD src homology 2 domain–containing leukocyte phosphoprotein), ADAP (adhesion and degranulation–promoting adaptor protein), SKAP-55 (55-kD src kinase–associated phosphoprotein) [3]–[5], as well as the GTP-binding protein Rap1, RapL (regulator of cell adhesion and polarization enriched in lymphoid tissues) and Riam (Rap1-GTP-interacting adapter molecule). Of these, SKAP-55 has a unique NH_2_-terminal region followed by a pleckstrin homology domain and a COOH-terminal SH3 domain [6]. It is expressed predominately in T cells and is needed for TcR induced ‘inside-out’ signaling that up-regulates LFA-1 clustering, adhesion and T cell–APC conjugation [7]–[10]. The SH3 domains of SKAP-55 and ADAP mediate reciprocal binding [11], [12]–[14], while the loss of the SH3 domain results in impaired LFA-1 adhesion [7]. Similarly, the loss or reduction of SKAP-55 expression resulted in an impairment of TcR induced LFA-1 clustering and adhesion [10]. Two-hybrid and over-expression studies have also reported binding to the phosphatase CD45 [15].

Despite its importance in adhesion, it has not been clear whether SKAP-55 can influence other signaling events in T-cells. In this respect, p21^ras^ operates upstream in the activation of extracellular signal-regulated kinase-1 and 2 (ERKs 1,2) [16], [17]. The cascade involves MAPK kinase kinase (MEK3) and MAPK kinase (MEK or MKK) [16], [17]. Ligation of the antigen-receptor on T-cells can activate p21^ras^ by means of either guanine nucleotide exchange factor (GEF), Son of sevenless (Sos) or Ras guanyl nucleotide releasing protein-1 (RasGRP1) [16], [18], [19], [20]. SOS participates by binding to Grb2 (growth factor receptor-bound protein 2) that in turn binds to the adaptor LAT (linker for activation in T cells) [5]. This probably represents a minor pathway. By contrast, RasGRP1 appears to predominate in p21^ras^ activation as shown with impaired T-cell activation and thymocyte development in deficient T-cells [21]–[24]. RasGRP1 is expressed primarily in T-cells and is comprised of a diacylglycerol (DAG)-binding C1 domain, an atypical pair of calcium-binding elongation factor (EF) hands and a catalytic domain with a p21^ras^ exchange motif. The C1 domain mediates membrane recruitment in response to phorbol ester and TcR activation [25]. Mutations in RasGRP1 have been linked to autoimmunity [26], while most p21^ras^ activation in T-cells in response to anti-CD3 ligation appears to occur in the trans-Golgi network [27], [28].

Here, we report the unusual finding that *SKAP-55−/−* primary T-cells and shRNA knock down (KD) T-cells have increased anti-CD3 induced ERK activation, concurrent with defective LFA-1 mediated adhesion. RNAi knock down (KD) of SKAP-55 in T-cell lines also showed an increase in p21^ras^ activation. SKAP-55 bound to the Ras guanine nucleotide exchange factor RasGRP1 in an SH3 dependent manner. Loss of RasGRP1 binding with SKAP-55ΔSH3 reversed SKAP-55 inhibition of ERK and ELK phosphorylation and ELK-dependent transcriptional activity. Lastly, *SKAP-55−/−* primary T-cells resulted in an increased presence of RasGRP1 in the trans-Golgi network where p21^ras^ becomes activated. These findings indicate that SKAP-55 has a negative regulatory role on the p21^ras^-ERK pathway, while positively regulating T-cell adhesion.

## Results

### ERK hyper-activation in SKAP-55 deficient T-cells

We have recently reported that the SKAP-55 deficient mouse shows major defects in T-cell adhesion [10]. Given this, we were surprised by the observation that T-cells with reduced or a loss of SKAP-55 expression showed a consistent amplification of the activation of extracellular receptor kinases (ERKs) in response to anti-CD3 ligation. Initially, SKAP-55+/+ and *−/−* T-cells were compared by staining with AlexaFluor647 labeled anti-pERK to the phosphorylation sites pThr185/pTyr187 followed by flow cytometric analysis. Mean fluorescent intensity (MFI) and percent of positive cells were measured. As shown in one representative experiment, anti-CD3 increased the MFI staining of SKAP-55+/+ T-cells from 6.53 to 8.58 (upper left panel; upper right histogram). Interestingly, the same stimulation increased pERK staining of *SKAP-55+/−* T-cells from 7.51 to 9.68 and *SKAP-55−/−* T-cells from 6.87 to 12.9 MFI (middle left panels; upper right histogram). The lower left panel shows a comparison of the different pERK staining patterns relative to that induced by pervanadate treatment. Further, enhanced pERK staining was more evident with the percentage of cells of positively stained cells. While anti-CD3 ligation increased the percentage of pERK positive wild-type cells from 4.79 to 17.64 percent of cells, the same treatment of *SKAP-55−/−* cells underwent a shift from 7.24 to 53.70 percent of cells (left panels; lower right panel). The difference between SKAP-55, *SKAP-55+/−* and *−/−* T-cells was consistently observed in more than seven experiments. Further, retroviral transduction of primary *SKAP-55−/−* T-cells restored the level of pERK staining to wild-type levels (data not shown).

**Figure 1:**
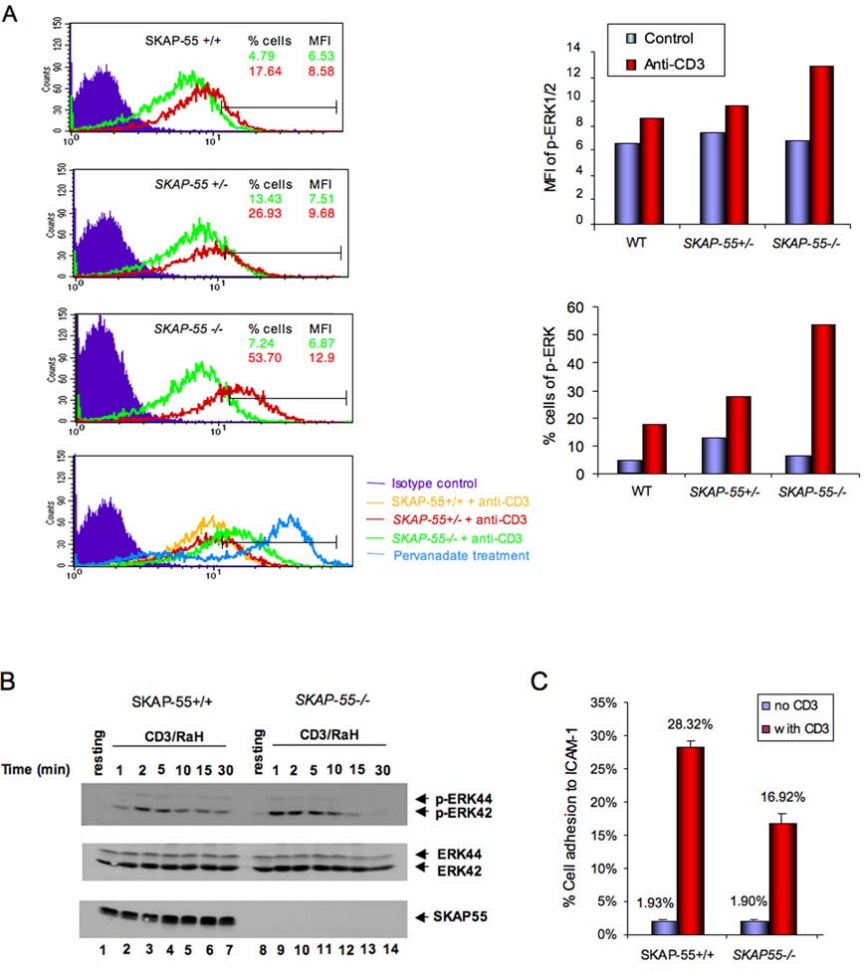
SKAP-55 deficient T-cells show enhanced anti-CD3 induced ERK activation. Panel A: FACS profile of T-cells from SKAP-55+/+, +/− and −/− mice stained with AlexaFluor647 labeled anti-pERK. T-cells from lymph-nodes were left unstimulated, or stimulated with anti-CD3-biotin (10 µg/ml) and streptavidin for 5 min. Upper panel: SKAP-55+/+; upper middle panel: *SKAP-55−/+;* lower middle panel: *SKAP-55−/−;* lower panel: comparison of anti-CD3 stimulated SKAP-55+/+, *SKAP-55+/−* and *SKAP-55−/−* cells vs. pervanadate treated cells. Right upper panel: histogram showing the difference in MFI values for SKAP-55+/+, *SKAP-55+/−* and *SKAP-55−/−* cells. Right lower panel: histogram showing the difference in the percentage of SKAP-55+/+, *SKAP-55+/−* and *SKAP-55−/−* cells staining for pERK. Panel B: Anti-pERK immunoblotting of anti-CD3 activated SKAP-55+/+ versus *SKAP-55−/−* T-cells. T-cells were activated with 5 µg/ml anti-CD3 for 1–30 minutes. Upper panel: anti-pERK blot; middle panel: anti-ERK blotting; lower panel: anti-SKAP-55 blot. SKAP-55+/+: lanes 1–7; *SKAP-55−/−:* lanes 8–14; Resting: lanes 1, 8; Stimulated: 1 min: lanes 2,9; 2 min: lanes 3, 10; 5 min: lanes 4,11; 10 min: lanes 5,12; 15 min: lanes 6, 13; 30 min: lane 7, 14. Panel C: SKAP-55 deficient T-cells have impaired adhesion to ICAM-1. Cells were stimulated with anti-CD3 followed by a measurement of binding to immobilized ICAM-1 on plates as described in *Material and Methods*.

Enhanced ERK phosphorylation in *SKAP-55−/−* CD4+ T-cells was observed with anti-CD3 ligation in a time-course followed by anti-pERK blotting (Figure 1B). Anti-CD3 ligation induced an increase in phospho-ERK from 1 to 10 min (lanes 2–5, 9–12) followed by a decrease in both *SKAP-55−/−* and +/+ T-cells by 15–30 minutes (lanes 13,14 vs. 6,7). *SKAP-55−/−* T-cells showed a 2–4 fold greater level of pERK staining from 1–10 minutes. Occasionally, the signal for *SKAP-55−/−* T-cells returned more rapidly to baseline levels than for wild-type cells, although this was not a consistent observation. As we recently reported [10], under the same conditions of anti-CD3 ligation, *SKAP-55−/−* T-cells showed a reduction in adhesion to ICAM-1 (Figure 1C). Overall, these observations indicate that the loss of SKAP-55 expression has opposing effects on anti-CD3 induced activation of T-cells, enhanced activation of ERK concurrent with a reduction in integrin adhesion of primary T-cells.

### Enhanced ERK activation in SKAP-55KD T-cells

**Figure 2 pone-0001718-g002:**
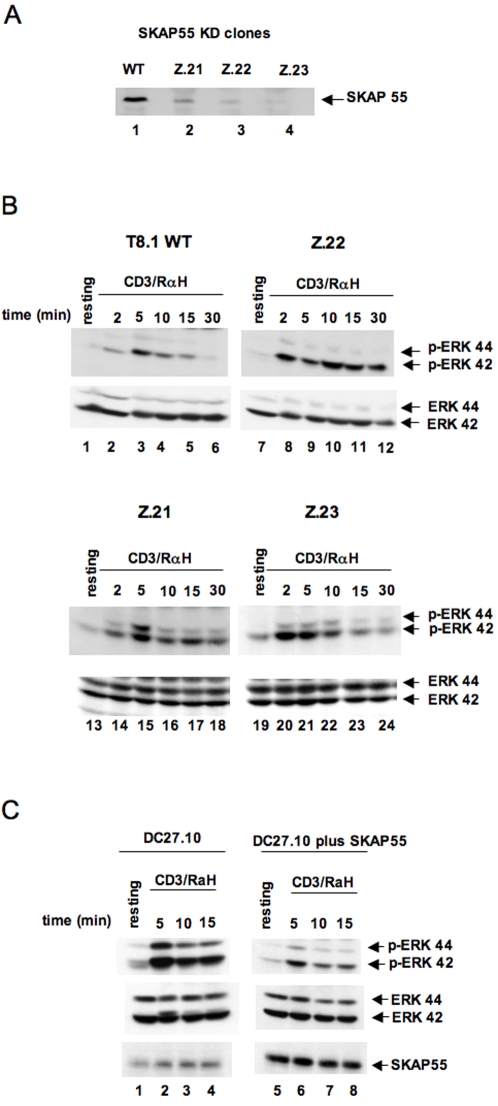
SKAP-55 knock-down and knock-up T-cells show altered ERK activation. Panel A: Upper panel: Reduction in SKAP-55 expression in SKAP-55KD cells. Z.21, Z.22 and Z.23 cell lines were derived from distinct SKAP55 shRNAs as described in *Material and Methods*. Panel B: Increased pERK in response to anti-CD3 in SKAP-55 KD cells. T8.1 WT and T8.1 SKAP-55 KD cells (Z.21, Z.22, Z.23) were either left untreated (lanes 1, 7,13, 19) or stimulated with anti-CD3 (10 µg/ml) (lanes 2–6; lanes 8–12; lanes 14–18; lanes 20–24). Upper panels: anti-pERK blotting; lower panels: anti-ERK blotting. Panel C: Reduction of ERK activation in DC27.10 cells. DC27.10 cells (lanes 1–4) and cells transfected with SKAP-55 (lanes 5–8) were either left untreated (lanes 1,5) or stimulated with anti-CD3 (5 µg/ml) for the indicated times (lanes 2–4 and 6–8). Upper panels: anti-pERK blotting; middle panels: anti-ERK blotting; lower panels: anti-SKAP-55 blotting.

To confirm this finding with a different system, shRNA clones (SKAP-55KDs) derived from T8.1 cells were used for analysis. Three independent cell lines (Z.21; Z.22; Z.23) were derived using distinct SKAP-55 shRNAs, as outlined in *Material and Methods*. Each shRNA was unique to SKAP-55. As shown in Figure 2A, each of the SKAP-55KD cells showed greatly reduced (i.e. >90 percent) SKAP-55 expression (lanes 2, 3, 4 vs. 1). No effect was noted on the expression of other proteins such as SKAP-55Related/Hom (data not shown; 8). Surface staining for anti-CD3 confirmed that the cells expressed similar levels of the TcR/CD3 complex (data not shown). Anti-pERK blotting confirmed the enhanced activation of ERK in the shRNA knock-down (KD) cells (Figure 2B). Anti-CD3 increased levels of phospho-ERK in WT cells over 2–10 minutes (Figure 2B). The same stimulation of Z.21, Z.22 and Z.23 cells showed a two to five fold higher level of pERK (lanes 2–4 versus lanes 8–10; 14–15; 20–22). The 42Kd form of ERK generally was affected more than the 44Kd form. For unknown reasons, a difference in the relative expression of the 44 versus 42Kd isoform was observed in different cell lines. However, this variation had no effect on the observed increase in pERK observed in SKAP-55KD cells. As a control, anti-ERK blotting showed similar levels of ERK (lower panels). These findings confirmed using three distinct SKAP-55 shRNA expressing cells that reduced SKAP-55 expression leads to an increase in ERK activation in response to anti-CD3 ligation.

Using a complementary third approach, SKAP-55 was over-expressed in DC27.10 cells followed by an assessment of ERK phosphorylation (Figure 2C). The expression of SKAP-55 markedly reduced anti-CD3 induced pERK over the 5–15 min time-course (upper panel, lanes 6–8 versus 2–4). Anti-ERK served as a control for ERK protein expression (middle panel). Anti-SKAP-55 blotting confirmed the increased expression of SKAP-55 (lanes 5–8 versus 1–4). Therefore, using the reverse approach, over-expression of SKAP-55 confirmed its inhibition for ERK activation in T-cells.

### Enhanced p21^ras^ activation in SKAP-55KD cells

**Figure 3:**
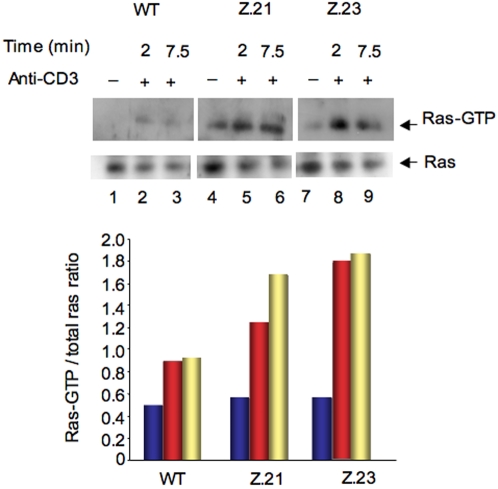
Hyper-activation of p21^ras^ in SKAP-55 KD cells. Upper panel: WT, Z.21 and Z.23 cells were ligated with anti-CD3 for various times and lysates were subjected to a pull-down assay using GST-Raf-1-RBD and blotted with Ras specific antibody. Middle panel: Anti-Ras blotting of cell lysates. Lanes 1–3: WT; lanes 4–6: Z.21; lanes 7–9: Z.23. Untreated: lanes 1,4,7; anti-CD3 ligation: 2 min: lanes 2,5,8; 7.5 min: lanes 3,6,9. Lower panel: Histogram showing the relative intensity of signal in the blot.

The regulatory effect of SKAP-55 on ERK activation could be due to an effect upstream on p21^ras^. To assess whether SKAP-55 influences p21^ras^, WT, Z.21 and Z.23 KD cells were cross-linked with anti-CD3 and assessed for GTP-bound p21^ras^ in a pull-down assay using GST-Raf-1 binding domain (RBD) fusion protein (Figure 3). While anti-CD3 increased the level of active p21^ras^ in WT cells at 2 and 7.5 min (upper panel, lanes 2,3 vs. 1), Z.21 and Z.23 KD cells showed higher levels of p21^ras^ at both time points (lanes 5,6; 8, 9 vs. 2,3). As a control, the lower panel shows levels of Ras in the different cells. The level of Ras expression was found to decrease slightly in response to anti-CD3 activation in wild-type and KD cells. Normalized RasGTP/Ras ratios showed a significant increase in Ras-GTP occupancy in Z.21 and Z.23 relative to wild-type cells (lower histogram). These observations indicated that SKAP-55 directly alters the activation of p21^ras^, the upstream regulator of the ERK pathway.

### SKAP-55 binds to RasGRP1

**Figure 4:**
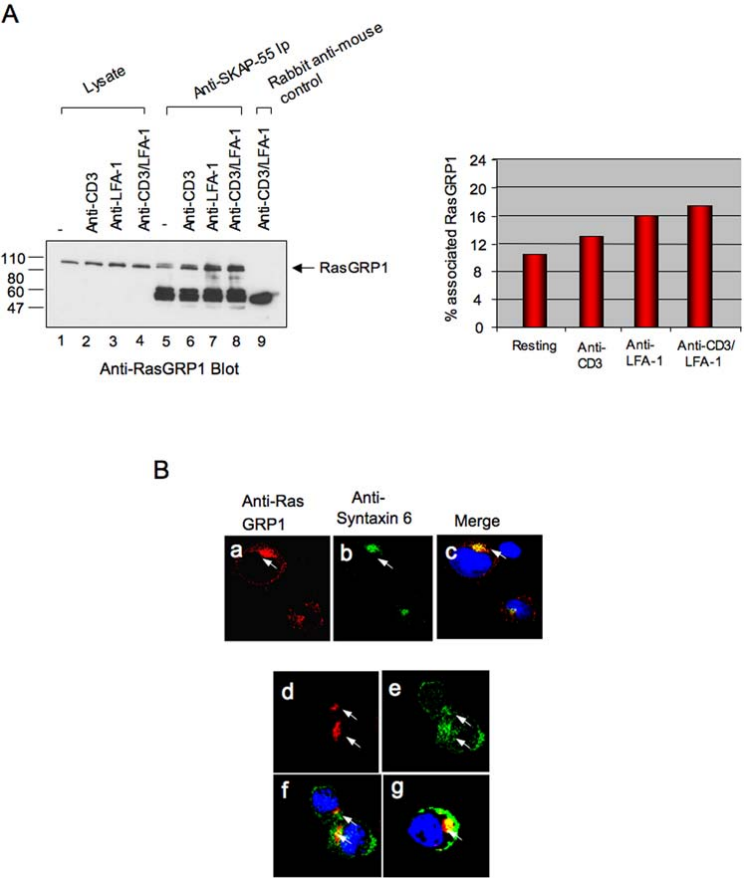
SKAP-55 associates with a subset of RasGRP1 and co-localizes with RasGRP1 in the TGN. Panel A: SKAP-55 binds to RasGRP-1. DC27.10 T-cells were stimulated with anti-CD3 or anti-CD18 (LFA-1) for 15 minutes. Cells were then lysed and subjected to precipitation with anti-SKAP-55 followed by anti-RasGRP1 blotting (lanes 1–9). Lanes 1–4: cell lysates; lane 9: rabbit anti-mouse control. Right panel: histogram showing the percent of RasGRP1 associated with SKAP-55 in resting and stimulated cells. Panel B: SKAP-55 partially co-localizes with RasGRP-1 in the TGN of DC27.10 cells. Cells were stained with anti-rabbit RasGRP-1/anti-rabbit AlexaFluor568 and anti-mouse Syntaxin6/anti-mouse AlexaFluor647 or anti-mouse SKAP-55/anti-mouse Alexa647. Upper panels: RasGRP-1 staining occurs in the TGN. Panel a: anti-RasGRP-1; panel b: anti-syntaxin 6; panel c: anti-RasGRP1/Syntaxin. Lower panels: panel d: anti-RasGRP1; panel e: anti-SKAP-55; panel f; anti-SKAP-55/RasGRP-1; panel g: anti-SKAP-55/RasGRP-1 staining of activated C57BL/6 primary T-cells. For analysis, at least 100 cells were counted.

Given that p21^ras^ was affected by SKAP-55, the next question concerned the identity of the upstream regulator. The main regulator of Ras in T-cells is the guanine nucleotide exchange factor RasGRP1 [22]–[24]. To assess initially whether SKAP-55 interacts with RasGRP1, Jurkat cells were ligated with anti-CD3, anti-LFA-1 or anti-CD3/LFA-1 followed by an anti-SKAP-55 precipitation and anti-RasGRP1 blotting (Figure 4A). Significantly, anti-SKAP-55 precipitated a band at 90Kd that was recognized by anti-RasGRP1 (lanes 5–8) and co-migrated with a band recognized in cell lysates (lanes 1–4). Rabbit anti-mouse served as a negative control (lane 9). Anti-CD3 ligation increased the level of precipitated RasGRP-1 (lane 6), as did anti-LFA-1 (lanes 7) and a combination of anti-CD3/LFA-1 (lane 8). Blotting with anti-SKAP-55 showed equal levels of SKAP-55 in each of the lysates for immunoprecipitation (data not shown). No co-precipitation was observed with other p21^ras^ regulators such as SOS or 3CG (data not shown). An estimate of 10–12% of total cellular RasGRP1 was found associated with SKAP-55 in resting cells based on a comparison of the intensity of signal in lysates versus immunoprecipitates as normalized against cell numbers (right histogram). This increased to 13–17 percent in response to anti-CD3 and LFA-1 ligation (right histogram).

Consistent with this, immunofluoresence microscopy showed a partial overlap between anti-SKAP-55-AlexaFluor488 and anti-RasGRP1-AlexaFluor568 staining patterns in DC27.10 T-cells (Figure 4B). As previously reported [21]–[24], RasGRP1 of resting cells localized predominately in the trans-Golgi network (TGN) as stained with anti-Syntaxin (panel a vs. b; panel c shows overlap). This contrasted with the broader anti-SKAP-55 staining that partially overlapped with the RasGRP1 staining (panel e vs. d; panel f shows overlap) and was also found in other regions of the cytoplasm and/or near the plasma membrane (panels e and f). Over four experiments, a range of 15–30 percent of stained SKAP-55 overlapped with the RasGRP1 pattern. This partial RasGRP1-SKAP-55 co-localization was also observed in activated primary T-cell blasts (panel g). Overall, using the approaches of biochemistry and microscopy, our findings are consistent with the notion that a subset of SKAP-55 and RasGRP1 molecules interact with each other.

### Disruption of SKAP-55 binding to RasGRP1 prevents adaptor mediated inhibition of pERK pathway

**Figure 5:**
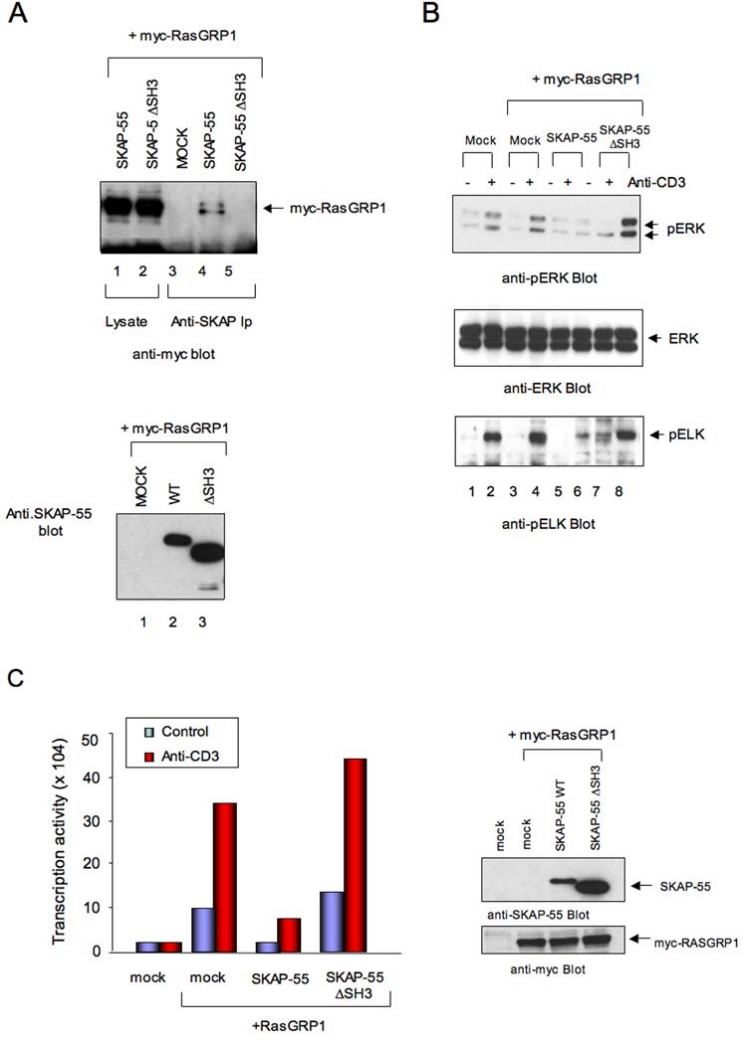
Loss of the RasGRP1 binding region in SKAP-55 abrogates its ability to inhibit ERK activation and ELK transcriptional activity. Panel A: RasGRP1 binding to SKAP-55 depends on the C-terminal domain of SKAP-55. Upper panel: deletion of the SKAP-55 SH3 domain prevents RasGRP1 binding. Lanes 1,2: myc-RasGRP1 expression in Jurkat cells transfected with SKAP-55 and SKAP-55ΔSH3. Lanes 3–5: association of RasGRP1 with SKAP-55 depends on the C-terminal domain of SKAP-55. Cell lysates: Lanes 1,2; anti-SKAP-55 precipitates: lanes 3–5. SKAP-55 transfection: Lanes 1, 4. SKAP-55ΔSH3 transfection: Lanes 2, 5. Lower panel: anti-SKAP-55 blot. Lane 1: transfection with myc-RasGRP1; lane 2: transfection with myc-RasGRP1 and SKAP-55; lane 3: transfection with myc-RasGRP1 and SKAP-55ΔSH3. Panel B: Loss of the RasGRP1 binding region in SKAP-55 abrogates its ability to inhibit ERK and ELK activation. Upper panel: anti-pERK blot of lysates from cells transfected with myc-RasGRP1 plus SKAP-55 or SKAP-55ΔSH3. Resting cells: lanes 1,3,5,7; anti-CD3: lanes 2,4,6,8. Middle panel: anti-ERK blot; Lower panel: anti-pELK blot. Panel C: Loss of the RasGRP1 binding region in SKAP-55 abrogates its ability to inhibit ELK transcriptional activity. Left panel: histogram showing inhibition of ELK transcription activity by SKAP-55 and lack of inhibition by SKAP-55ΔSH3. Right panel: anti-SKAP-55 (upper) and anti-myc (myc-RasGRP1) blot of cell lysates.

Mapping studies showed that the deletion of the C-terminal region of SKAP-55 (i.e. SH3 domain) eliminated binding to RasGRP1 (Figure 5A, upper panel, lanes 4 vs. 5). Anti-SKAP-55 blotting confirmed expression of the SKAP-55 and SKAP-55ΔSH3 constructs (lower panel, lanes 2,3). This effect does not reflect a loss of SH3 domain function since mutation of key WW residues in the SH3 domain that are needed for SH3 domain binding did not interfere with binding between SKAP-55 and RasGRP1 (data not shown). Instead, the C-terminal region mediates binding via another as yet established mechanism. To assess the influence of SKAP-55-RasGRP1 interaction on the ERK pathway, proteins were co-expressed and assessed for effects on ERK activation and its downstream target, the ETS transcription factor ELK (Figure 5B). ELK is a well-established substrate and a downstream target of ERK [29]. Anti-CD3 increased both ERK (upper panel, lane 2 vs. 1) and ELK phosphorylation (lower panel, lane 2 vs. 1). Myc-RasGRP1 expression resulted in a weak increase in ERK and ELK phosphorylation (lane 4 vs. 2). Significantly, while SKAP-55 expression strongly inhibited anti-CD3 induced ERK and ELK phosphorylation (upper and lower panels, lane 6 vs. 4), the RasGRP1 binding mutant SKAP-55ΔSH3 failed to reduce phosphorylation (upper and lower panel, lane 8 vs. 4). In fact, in some experiments, SKAP-55ΔSH3 even increased ERK phosphorylation (upper panel, lane 8 vs. 6).

Consistent with this observation, SKAP-55 vs. SKAP-55ΔSH3 also differed in their effect on anti-CD3 plus RasGRP1 activation of a Gal-ELK promoter assay (Figure 5C). In this case, the expression of RasGRP1 markedly increased ELK driven promoter activity. This was seen using different concentrations of anti-CD3 and most clearly in response to sub-optimal (i.e. 0.25 µg/ml) concentrations of anti-CD3. As seen with the pERK and pELK assay, expression of SKAP-55 almost entirely (i.e. >95%) blocked the anti-CD3 driven ELK transcription activity (left histogram). By contrast, the expression of SKAP-55ΔSH3 failed to block and even potentiated the RasGRP1 effect further. Anti-SKAP-55 blotting confirmed the expression of SKAP-55 and SKAP-55ΔSH3 (right upper panel) and of myc-RasGRP1 (right lower panel). Although further analysis will be needed to define the exact basis of RasGRP1-SKAP-55 binding, these findings show that the loss of RasGRP1 binding correlated with the loss in the ability of SKAP-55 to suppress the ERK pathway.

### SKAP-55−/− T-cells show enhanced RasGRP1 localization in the TGN

**Figure 6:**
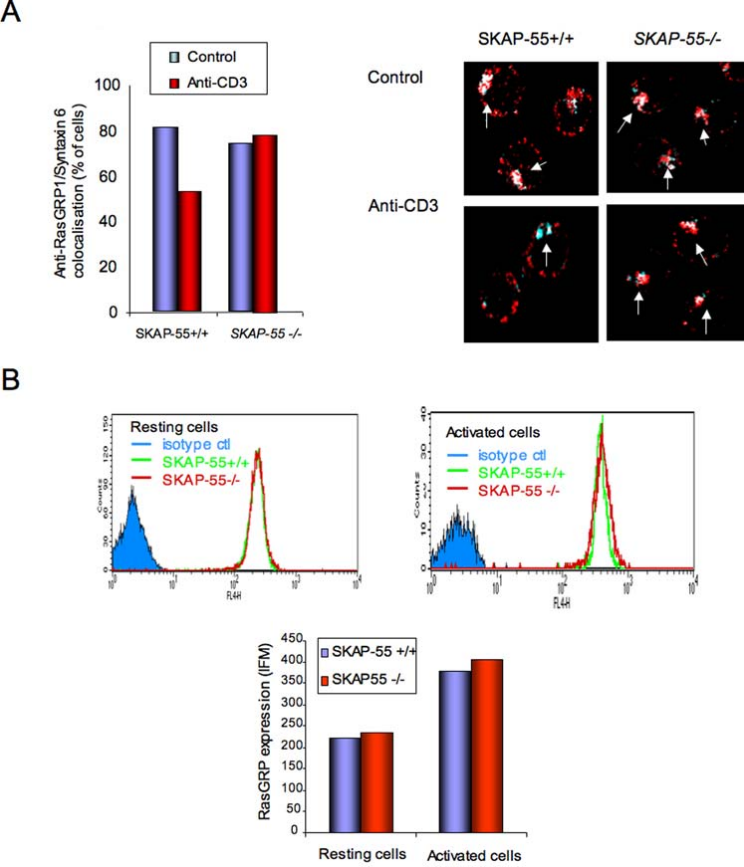
Increased RasGRP1 in the trans-Golgi network of *SKAP-55−/−* T-cells. Left panel: histogram showing % of T-cells expressing RasGRP-1 in the TGN (i.e. co-localization with Syntaxin) in SKAP-55+/+ versus SKAP-55−/− T-cells. Right panel: images of cells showing co-localization of RasGRP-1and TGN under resting and anti-CD3 activated conditions. For analysis, at least 100 cells were counted. Panel B: *SKAP-55 +/+* and *SKAP-55−/−* T-cells show equal amounts of RasGRP1. Upper panel: Cells were stained with anti-RasGRP1/anti-rabbit AlexaFluor647 and analysed by FACS. Lower panel: histogram showing the MFI values of *SKAP-55 +/+* and *SKAP-55−/−* T-cells.

Studies have shown that Ras is activated preferentially in the Golgi apparatus of T-cells in response to anti-CD3 [20], [28]. Since SKAP-55 can bind to RasGRP1, the regulator of p21ras in T-cells, we addressed whether there was any difference in the localization of the exchange factor in the Golgi of *SKAP-55−/−* and *+/+* T-cells in response to anti-CD3 ligation. An increased RasGRP1 localization in the TGN might therefore explain the enhanced activation of the ERK pathway observed in *SKAP-55−/−* T-cells. To this end, cells were either left untreated or ligated with anti-CD3 for 15 min followed by the staining of RasGRP1 with anti-RasGRP1 coupled to Alexa Fluor568, while the TGN was stained with anti-Syntaxin coupled to Alexa Fluor647. RasGRP1 co-localized with anti-Syntaxin staining of the trans-Golgi with both *SKAP-55−/−* and +/+ T-cells (left histogram and right upper panels). Interestingly, anti-CD3 stimulation reduced the number of cells with co-localized RasGRP1 in *SKAP-55+/+* T-cells from 80 to 55 percent of *SKAP-55+/+* T-cells. Examples of the co-localized images are shown in left panels. By contrast, *SKAP-55−/−* T-cells resulted in the maintenance of RasGRP1 in the TGN following anti-CD3 activation (i.e. from 75 to 78 percent of cells) (left histogram). The percentage of cells with TGN localized RasGRP1 was the same as in resting cells. This was observed in four independent experiments. Further, this was not the result of an overall increase in RasGRP1 expression in one group of cells versus another since anti-RasGRP1 analysis by FACS confirmed comparable levels of RasGRP1 (Figure 6B). SKAP-55+/+ and −/− cells also showed not difference in RasGRP1 levels in activated T-cells. *SKAP-55−/−* T-cells therefore differ from SKAP-55+/+ T-cells with the presence of greater levels of RasGRP1 in the TGN of T-cells following anti-CD3 ligation. This appears to result from the reduced exit or loss of RasGRP1 from the TGN in SKAP-55−/− T-cells relative to WT cells. Anti-CD3 induced Ras activation has been reported to occur in the TGN of T-cells [27], [28]. Overall, our observations demonstrate that SKAP-55 binds to RasGRP1 and affects the localization of RasGRP1 in the TGN where p21ras activation occurs. Although this observation does not prove cause and effect, the increased presence of RasGRP1 in the TGN in *SKAP-55−/−* cells is consistent with the increased activation of the ERK pathway in these cells.

## Discussion

While SKAP-55 has been established as a mediator of integrin adhesion in T-cells, an outstanding question has been whether the adaptor can regulate other aspects of T-cell signaling and function. In this way, SKAP-55 could act as a node to integrate or modify signals for adhesion (i.e. conjugation) and TCR signaling. This would also be in keeping with the observation that other adaptors such as SLP-76 and ADAP can regulate more than one event in cells. Distinct regions of SLP-76 can regulate cytokine production versus adhesion [30], while ADAP has recently been connected to the NFκB pathway [31]. In this study, we have identified a novel function of SKAP-55 in the negative regulation of the p12ras-ERK pathway in T-cells, in addition to its positive regulation of LFA-1 adhesion. *SKAP-55−/−* primary T-cells showed an increase in TCR mediated ERK activation, while the use of multiple independent shRNA knock down (KD) T-cells showed an increase in TCR mediated ERK and p21^ras^ activation. Conversely, SKAP-55 over-expression inhibited ERK phosphorylation at pThr185/pTyr187 and the activation of its downstream target ELK. In turn, the loss of RasGRP1 binding to the C-terminus of SKAP-55 abrogated the ability of SKAP-55 to inhibit ERK/ELK phosphorylation and ELK-dependent transcriptional activity. Lastly, *SKAP-55−/−* primary T-cells showed an increase in RasGRP1 in the trans-Golgi network in response to anti-CD3 ligation, the location where p21^ras^ is primarily activated in T-cells. These findings indicate that SKAP-55 has a dual regulatory function of enhancing LFA-1 mediated adhesion, while suppressing or raising the threshold needed for the activation of the ERK pathway.

The finding that SKAP-55 suppresses the p21^ras^-ERK pathway was observed in models that included primary SKAP-55 deficient T-cells, the Jurkat cell line, the DC27.10 hybridoma and the use of three independent shRNAs to knock-down SKAP-55 expression. The loss of SKAP-55 by genetic ablation or the reduction in expression by shRNA consistently resulted an increase in anti-CD3 induced ERK activation, and an increased RBD binding to p21^ras^, while the over-expression of SKAP-55 suppressed ERK activation (Figs 1–3). Intriguingly, these effects occurred under conditions where an opposing effect on LFA-1 clustering and adhesion was observed (Fig. 1). Reduced or a loss of SKAP-55 expression caused an impairment of LFA-1 adhesion (Fig. 1) [10], while increased SKAP-55 expression enhanced adhesion [7]–[10]. This points to a highly unusual situation in T-cells where a single mediator has opposing effects on p21^ras^-ERK activation and adhesion. To our knowledge, this opposing dichotomy in T-cells has not been previously reported. What might therefore be the purpose of these opposing effects? Could it be related to a need for dampening TCR signalling during the adhesion of T-cells, or a requirement for reduced adhesion during T-cell activation? One possibility could relate to the temporal regulation of TCR signaling events during T-cell/APC conjugation, or the migration of T cells along vessel surfaces where they exit into lymph nodes and/or other tissues. Lymphocytes use LFA-1 that binds to ICAMs expressed by APCs and on epithelial cells lining the blood vessels. In the context of conjugation, contact between T-cells and APCs is initially induced by chemokines followed by TCR induced ‘inside-out’ signals that leads to a major increase in the number of TCR-MHC peptide interaction events. The dampening of p21^ras^-ERK activation during an increase in adhesion and conjugation induced by SKAP-55 could alter the threshold of T-cell activation. In the context of certain MHC-peptide interactions, this could prevent the generation of partial activation signals that leads to anergy induction or non-responsiveness. Similarly, it could dampen the activation process during the initial contact period between T-cell and APC, before the T-cell has had an opportunity to assemble sufficient TCR-MHC interactions to produce a sufficiently strong signal needed for activation. By concurrently suppressing ERK activation, it would guard against incomplete or partial signaling linked to the induction of anergy [32]–[34]. Impaired ERK activation has also previously been shown to participate in anergy [34]. Likewise, SKAP-55 may act to prevent responses to low affinity ligand such as auto-antigen. Lastly, it may also operate to reduce integrin activation of the ERK pathway in the absence of TcR stimulation. In this context, VLA-4 ligation has been shown to activate ERK [35]. In the same vein, SKAP-55 may act to prevent inappropriate T-cell responses during the migration of T cells along vessel surfaces where they exit into lymph nodes and/or other tissues. Further studies will be needed to test this hypothesis.

The suppression of ERK activation could have resulted from the direct regulation of ERKs or their upstream regulator p21^ras^. Although p21^ras^ was not assayed in SKAP-55−/− T-cells, shRNA knock-down of the adaptor in cell lines showed an increased activation of p21^ras^ (Fig. 3). The increased p21^ras^ activation was also frequently observed in resting T-cells. This pointed to a connection with a regulator of p21^ras^ that in turn led to the identification of SKAP-55 binding to RasGRP1 (Fig. 4). During the preparation of this paper, the same observation was independently made by another group [36]. This would fit nicely in a scheme where SKAP-55 binding reduces the guanine nucleotide exchange activity of RasGRP1 and/or its intracellular localization in the cell. Binding to other GEFs such as SOS or 3CG was not observed (data not shown). RasGRP1 GEF activity is difficult to measure and so we have initially focused on the effect of the loss of RasGRP1 binding on SKAP-55 inhibition of ERKs and its localization in cells (Fig. 6). The loss of binding to the C-terminus of SKAP-55 resulted in a concurrent loss of RasGRP1 binding and its suppression of ERK and ELK (Fig. 5). This has provided initial evidence to link the two events. The one complication is that mutations of WW residues of the SH3 domain that are essential for SH3 domain function did not interfere with RasGRP1/SKAP-55 binding (data not shown). Therefore, instead of classic SH3 domain binding, there is a requirement for the C-terminal region for the binding to RasGRP1 that includes residues that comprise an SH3 domain. In this way, the loss of residues in the C-terminus was used to connect RasGRP1 binding to a loss of SKAP-55 suppressor activity, a result consistent with the need for RasGRP1 binding in the regulator of ERK. Further work will be needed to clearly define in a more precise way the molecular basis of binding between these two proteins.

Our findings differ from Kosco *et al*. who found that over-expression of SKAP-55 increased TCR-dependent AP-1 transcriptional activity, while the knock-down of SKAP-55 decreased ERK phosphorylation [36]. Although the basis for this difference is not clear, it could be related to different cell conditions and levels of over-expression. The same study found different effects depending on levels of SKAP-55 over-expression. The strength of our study is in the consistency of findings using both *SKAP-55−/−* primary cells and over-expression or knock-down analysis in different cell lines. SKAP-55 deficient primary T-cells clearly showed an enhancement of ERK activation, and this was observed in our shRNA knock-down studies with cell lines. We also directly assessed the status of p21ras in our KD cells. In all cases, the loss or reduction in SKAP-55 expression resulted in enhanced p21ras or ERK activation.

Lastly, another observation that implicated RasGRP1 in the suppression ediated by SKAP-55 was the greater level of RasGRP1 in the TGN in anti-CD3 activated *SKAP-55−/−* primary cells (Fig. 6). Philips and coworkers have shown that the active form of p21^ras^ in T-cells induced by anti-CD3/TcR ligation is found in the Golgi [27], [28]. Although the molecular basis of the observation needs greater definition, it is compatible with the notion of greater p21ras activation in T-cells linked to a connection between SKAP-55 and RasGRP1. The increased presence of the GEF could be due to increased migration to the TGN, or more likely, a lesser degree of RasGRP1 movement from the TGN in response to TCR signals. Active p21ras has been reported in both the TGN and the plasma membrane, depending on the cell type examined. In Jurkat T-cells activated by anti-CD3 alone, active p21ras has been reported to remain in the TGN, while coligation with LFA-1 promotes more translocation to the plasma membrane [19], [20]. Our findings are compatible with the notion of a weaker TcR signalling or decoupling in SKAP-55 deficient T-cells. The net result was to maintain more RasGRP1 in the Golgi, the location where p21ras is activated in T-cells. We also observed an increase in the expression of Ras in the Golgi (data not shown). Overall, this would fit with the model where SKAP-55 plays a role in modulating the coupling of the TCR with the localization of RasGRP1, although this observation will require more work to define this clearly. RasGRP1 activates Ras with the involvement of diacylglycerol via phospholipase C-γ and actin polymerization [37], [38]. Future studies will be needed to assess whether SKAP-55 can act as a chaperone for the translocation of RasGRP1 to the plasma membrane. The PH domain of SKAP-55 could provide a mechanism to facilitate the movement of a portion of RasGRP-1 from different intracellular compartments.

## Materials and Methods

### Cells and Antibodies

Murine hybridoma T8.1, a kind gift from Dr. Oreste Acuto (Oxford University, Oxford, UK) were cultured in DMEM supplemented with 200 nM methotrexate, 1 mg/ml G418, 10 percent fetal calf serum (FCS), 10 mM Hepes, 2 mM L-Glutamine, 100 U/ml penicillin/streptomycin, and 5×10^−5^ M 2-mercaptoethanol. The generation of SKAP-55 knock-out mice has been described elsewhere [10]. Antibodies against SKAP-55, RasGRP1 and Ras were purchased from Transduction Laboratories (San Diego, CA); anti-Syntaxin 6 from BD Biosciences, anti-CD3ε (2C11; hamster anti–mouse CD3), and anti-CD11a (LFA-1 α-chain) or anti-CD18 (LFA-1 β-chain) from Pharmingen (Oxford, UK). Alexa Fluor546, Alexa Fluor633, AlexaFluor568 or AlexaFluor647 conjugated secondary Abs were purchased from Molecular Probes (Eugene, OR). Anti-phospho ERK1/2 T202/Y204 or T185/Y187 was purchased from Biosource (BioSource United Kingdom, Belgium).

### FACS staining and Immunofluorescence

For detection of phospho-ERK by flow cytometry, cells were permeabilized, stained with AlexaFluor647 tagged anti-phospho-ERK1/2 and analyzed by FACS (BD FacsCalibur), as described [39]. For immunofluoresence, resting and cells stimulated with anti-CD3 (5 µg/ml; 15 min) were fixed in 4% PFA, permeabilized with 0.3% saponin and stained with anti-RasGRP-1, anti-Ras, anti- SKAP-55, anti-Syntaxin 6 and appropriate fluorochrome-labelled secondary antibody, respectively. For nucleus staining, Hoechst was added together with secondary antibodies. Negative controls were run with unstained, single stained samples or without primary antibody. Stained cells were mounted on slides and analyzed by confocal microscopy.

### Generation of T8.1 SKAP-55 knock down (KD) cells

T8.1 cells were transfected with three different shRNA SKAP-55 oligonucleotide pairs which were ligated into the BbsI sites of the psiRNA-hH1 vector (InvivoGen, San Diego, CA): 5′ACCTCATAACGTAATCAAGCAAGGATTCAAGAGATCCTTGCTTGATTACGTTATTT 3′ and 5′CAAAAAATAACGTAATCAAGCAAGGATCTCTTGAATCCTTGCTTGATTACGTTATG 3′ (resulting clones termed Z.21); 5′ACCTCAGGACAGCTCTGATGATAATCTCAAGAGGATTATCATCAGAGCTGTCCTTT 3′ and 5′ CAAAAAAGGACAGCTCTGATGATAATCCTCTTGAGATTATCATCAGAGCTGTCCT 3′ (resulting clones termed Z.22); 5′ ACCTCATAACGTAATCAAGCAAGGATTCAAGAGATCCTTGCTTGATTACGTTATTT 3′ and 5′CAAAAAATAACGTAATCAAGCAAGGATCTCTTGAATCCTTGCTTGATTACGTTATG 3′ (resulting clones termed Z.23). The transfected cells were cultured in Zeocin containing medium and stable clones were screened for the loss or reduction of SKAP-55 greater than 90% and unaffectiveness of other proteins as described [8].

### Immunoblotting

DC27.10 cells transiently transfected with pSRαSKAP-55, T8.1 SKAP-55 KD cells and primary SKAP-55 KO cells were stimulated with anti-CD3 (5 µg/ml for DC27.10 and SKAP-55KO cells; 10 µg/ml for T8.1SKAP-55KD cells) and rabbit anti-hamster (half the concentration of primary mAb) antibodies for various times. Cells then were lysed and subjected to immunoblotting with anti-phospho-ERK1/2 and total ERK1/2. The levels of bound antibody were measured using HRP-conjugated anti-mouse/rabbit antibodies followed by detection with enhanced chemiluminescence. Stimulation experiments with anti-CD3 or anti-LFA-1 and immunoblotting were conducted as described [40].

### Ras activation assay

For the Ras activation assay, T8.1SKAP-55 KD cells were stimulated with anti-CD3 (10 µg/ml) and rabbit anti-hamster (5 µg/ml) antibodies for various times. Activated Ras was isolated from stimulated cell lysates using agarose-coupled GST-Raf1-RBD (Upstate Biotechnology) as described [41]. Complexes were analyzed by SDS-PAGE and immunoblotting with Ras-specific antibody. The densitometric analysis of the bands was performed using NIH Image software.
